# Trends of contraception among ladies of local population in Pakistan; why, how, when and what?

**DOI:** 10.12669/pjms.323.9662

**Published:** 2016

**Authors:** Khaula Atif, Afeera Afsheen, Syed Abid Hassan Naqvi, Saleem Asif Niazi, Habib Ullah Khan

**Affiliations:** 1 Dr. Khaula Atif, MBBS, MCPS (Family-Medicine), Department of General Administration, Combined Military Hospital, Peshawar Cantonment, Khyber Pakhtun Khwah, Pakistan-54000; 2 Dr. Afeera Afsheen, MBBS, MCPS (Gynecology), FCPS (Gynecology). Department of Gynecology, Combined Military Hospital, Peshawar Cantonment, Khyber Pakhtun Khwah, Pakistan-54000; 3 Dr. Syed Abid Hassan Naqvi, MBBS, FCPS, FRCS, MS(Ophthalmology). Department of Ophthalmology, Combined Military Hospital, Peshawar Cantonment, Khyber Pakhtun Khwah, Pakistan-54000; 4 Dr. Saleem Asif Niazi, MBBS, MCPS(ENT), FCPS(ENT). Department of ENT, Combined Military Hospital, Peshawar Cantonment, Khyber Pakhtun Khwah, Pakistan-54000; 5 Dr. Habib Ullah Khan, MBBS, FCPS (General Surgery), FCPS (Neurosurgery). Department of Neurosurgery, Combined Military Hospital, Abbotabad Cantonment, Khyber Pakhtun Khwah, Pakistan

**Keywords:** Family Planning, Contraceptive Methods, Contraceptive Devices, Under-developed countries

## Abstract

**Objective:**

To analyze trends of use of methods of contraception along with study of impact of various demographic and social factors on contraception in Peshawar, Pakistan.

**Methods:**

A cross-sectional descriptive study with random purposive sampling was conducted at Combined Military Hospital Peshawar, from Mar 2015-Nov 2015. Self-designed questionnaire with demographic details and questions pertinent to contraceptive practices was utilized as study instrument. Females reporting to concerned hospital for contraceptive advice and prescription were distributed with questionnaire and written informed consent form. Formal approval was taken from ethical committee of hospital. Data was analyzed via descriptive analysis (SPSS-21), qualitative data was expressed as frequencies and percentages; quantitative as mean±standard deviation (SD). Main outcome variable i-e contraceptive device used; was cross-tabulated with independent variables.

**Results:**

Response rate was 53.2% (n-426). Usage of contraceptive device was as follows; 51.2% Nil, 9.4% barriers, 22.3% oral/injectable hormones, 13.4% IUCDs, 3.8% sterilization. There was a strong relationship between type of contraceptives used and age (*p<0.001*), client’s education (*p<0.001*), husband’s education (*p<0.001*), number of children (*p<0.001*), religion (p0.013), socioeconomic class (*p<0.001*), and religious beliefs about use of contraceptives (*p<0.001*). More Muslims considered contraception irreligious than non-Muslims (*p* 0.02). There was no significant impact of husbands’ pressure to not to use contraceptives on type of contraception practised (*p 0.114*).

**Conclusion:**

Contraceptive devices are under-utilized in the study participants. Multidisciplinary approach should be applied to enhance client education, awareness and counseling to utilize these devices more appropriately and regularly.

## INTRODUCTION

Today’s world is neither unaware of the term “Family Planning” nor defies its importance. Nevertheless, “Contraception” remains misunderstood, misperceived or misused; especially in under-developed countries. Family planning was declared one of the vital MDG (Millennium Development Goals).^1^ Lack of knowledge and misconceptions are major impediments in use of contraceptives^2^ more so in Asia.^1^ Almost 30% of pregnancies are unintended in the world, validating lack of apt contraception,^3^ leading to illegal/criminal abortions, unwelcomed deliveries and challenges to maternal health (physical and mental).^1^

In Asia, the first ever family planning program was launched by Pakistan, but ironically it is overtly neglected and under-utilized.^4^ Many factors adversely affect adequate use of contraceptives like religious/social believes or economical constraints. Literature states that despite free availability, 58% people avoid using contraceptives.^5^ Pakistan faces equal challenges. With a multitude of social/traditional zones, considerable variation is observed in contraceptive practices. Peshawar is a metropolitan city and provincial capital, where religious and social traditions at times dominate the educational beliefs. As per the meager knowledge of authors, no detailed study has yet been carried out in this region to analyze trends of use of contraceptive devices along with impact of demographic, religious and social factors on its prevalence. Randomly selected cohort fairly represents the population of area and can give appreciable results.

Our objective was to analyze trends of use of methods of contraception along with study of impact of various demographic and social factors on contraception in Peshawar, Pakistan.

## METHODS

It was a cross-sectional descriptive study; with random purposive sampling. A self-designed questionnaire with demographic details and questions pertinent to contraceptive practices was utilized as study instrument. Subjects were ladies reporting to hospital for contraceptive advice and prescription. Formal approval was taken from ethical committee of the hospital and written informed consent from the participants.

Keeping the margin of error, confidence level and response distribution 5%, 95% and 50% respectively, recommended sample size was 385 to represent a 3.5 million population of concerned city. The prior estimation of non-response rate was 50%, therefore, 800 questionnaires were distributed to volunteers. Data analysis was done via descriptive analysis (SPSS-21), qualitative data expressed as frequencies and percentages; quantitative as mean±standard deviation (SD). Main outcome variable i-e contraceptive device used; was cross-tabulated with independent variables (age, client’s education, husband’s education, number of children, religion, socioeconomic class, religious beliefs and husband’s pressure about contraception).

## RESULTS

The response rate was 53.2%(n-426). Mean age was 32.36±6.821 years (Minimum 22 & Maximum 50); Client’s Education 6.30±7.196 years (Minimum Nil & Maximum 23); Education of Client’s Husband 10.18±5.897 (Minimum Nil & Maximum 24) and Number of Children 3.44±2.076(Minimum Nil & Maximum 13); 100% respondents were married, all were either Muslim or Christian. Frequencies of qualitative variables and percentages of use of contraceptive devices among different groups are highlighted in Table-I & II respectively.

**Table-I:** Frequencies of qualitative variables of study participants (n-426).

Age Group (Years)	22-29(37.6)	30-39 (43.2)	≥40(19.2)	-	-	-
Client’s education	Group-I^1^(51.2)	Group-II^2^ (20.7)	Group-III^3^(18.8)	Group-IV^4^(9.4)	-	-
Husband’s education	Group-I^1^(18.8)	Group-II^2^(39.4)	Group-III^3^(27.7)	Group-IV^4^(14.1)	-	-
Number of Children	Nil (5.6)	1-2 (24.2)	3-4 (51.4)	5-7 (12.0)	≥8 (6.8)	-
Religion	Islam (92.5)	Christian (7.5)	Others (0.0)	-	-	-
SEC^5^	Low (65.0)	Middle (30.0)	Upper (4.9)	-	-	-
Contraceptive Used	Nil (51.2)	Barriers (9.4)	Oral/Injectable Hormones (22.3)	IUCDs (13.4)	Sterilization (3.8)	-
Is it Irreligious to Use Contraceptives	No (57.5)	May Be (7.3)	Yes (35.2)	-	-	-
Husbands’ Pressure to not to Use Contraceptives	No (88.7)	May Be (0.2)	Yes (11.0)	-	-	-

1 Nil.

2 Primary-Matriculate.

3 Intermediate- Masters.

4 Professional Education- Consultant.

5 Socioeconomic Class.

**Table-II:** General trends of use of contraceptives among study participants.

*Variable*		*Most Frequently*	*Second Most Frequent*	*Least*
Age (Years)	20-29	Nil	Hormones^1^	Sterilization
	30-39	Nil	IUCDs	Sterilization
	≥40	Nil	Hormones	Barriers
No of Children	Nil	Nil	Barriers	Sterilization
	1-2	Nil	Hormones	Sterilization
	3-4	Nil	Hormones	Sterilization
	5-7	Nil	Hormones	Barriers
	≥8	Nil	Sterilization	Barriers
Client’s Education	Group-I^2^	Nil	Hormones	Barriers
	Group-II^3^	Nil	Hormones	Sterilization
	Group-III^4^	Nil	Hormones	Sterilization
	Group-IV^5^	IUCDs	Hormones	Nil
Husband’s Education	Group-I^2^	Nil	Hormones	Barriers
	Group-II^3^	Nil	Hormones	Sterilization
	Group-III^4^	Nil	IUCDs	Sterilization
	Group-IV^5^	IUCDs	Barriers	Sterilization
Religion	Islam	Sterilization	Hormones	Sterilization
	Christian	Hormones	Nil	Barriers
Socioeconomic Class	Low	Nil	Hormones	Sterilization
	Middle	Nil	Hormones	Sterilization
	Upper	IUCDs	Hormones	Barriers

1 Oral/Injectable Hormones.

2 Nil.

3 Primary-Matriculate.

4 Intermediate- Masters.

5 Professional Education- Consultant

**Fig.1:**
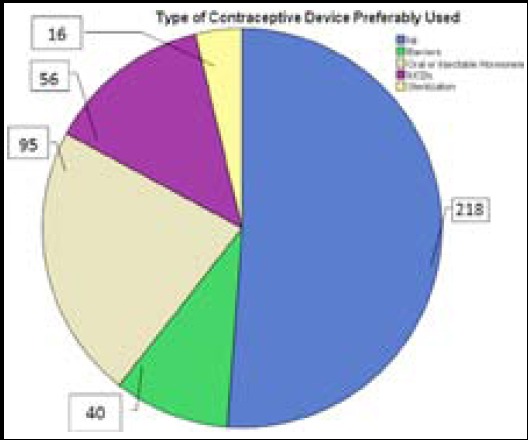
Trends of use of contraceptives among study participants (n-426).

Fig.1 shows frequencies of various contraceptive methods in vogue by respondents. There was a strong relation between type of contraceptives used and age (*p<0.001*), client’s education (*p<0.001*), husband’s education (*p<0.001*), number of children (*p<0.001*), religion (*p0.013*), socioeconomic class (*p<0.001*), and religious beliefs about use of contraception (*p<0.001*). More Muslims considered contraception irreligious as compared to non-Muslims (*p<0.02)* There was no significant impact of husband’s pressure to not to use contraception on outcome variable(*p 0.114*).

## DISCUSSION

Adequate maternal health can never be delinked from family planning, which whereby demands apt contraception.^1^ Majority of people in the world are aware of this term even if they have never practiced it.^1^ In this study 51.2% subjects were not using contraception of any type. Pakistani researchers documented that 16% people had never used any contraceptive; while 77% did practice some form of contraception regularly or otherwise.^6^ In Pakistan 26% limiters use modern contraceptive devices.^4^ International literature endorsed varying contraceptive prevalence; 89% in China being highest in 2010,^7^ followed by appreciable figures like 78.5% in USA^8^ and 60% in Zimbabwe.^9^ There has been a gradual but steady rise in contraception with declining fertility in the world; like in Zimbabwe^9^, Germany^10^ and Spain.^11^

**Fig.2:**
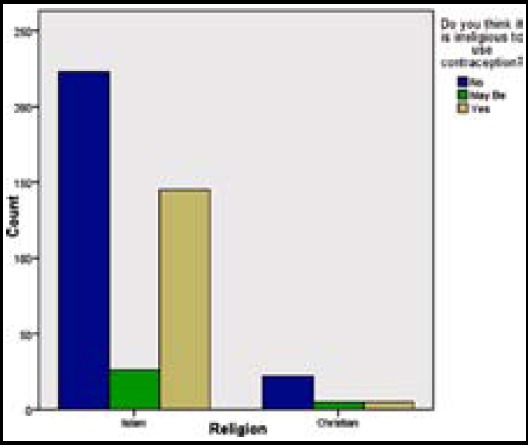
Impact of religious beliefs on use of contraceptive methods.

Various methods of contraception are available globally, some limiters avoid conception without devices (withdrawal/coitus-interruptus), others may prefer barriers (condoms/diaphragms), oral/injectable hormones, intrauterine contraceptive devices (IUCDs); or permanent sterilization (tubal-ligation/vasectomy In this study 9.4% used barriers, 22.3% oral/injectable hormones, and 13.4% IUCDs, while 3.8% had opted for permanent sterilization. The break-up of various preferences for contraception is endorsed in Table-II.

National, regional and global researches revealed diversified figures. Few stated withdrawal^12^,^13^ and condoms^4^-^6^,^13^-^16^ were the commonest methods; condoms used by 93%,^17^ 24.3%^16^ 79.9%,^18^ 24.3%,^16^ and 20.8%,^8^ while withdrawal by 60%^17^ and 38.9%.^18^

Regarding hormones; oral contraceptives (OCs)^5^,^19^,^20^ and injectable preparations (Depot medroxyprogesterone acetate/DMPA being the commonest)^17^,^19^,^20^ are considered effective and commonly used, their prevalence being 23%^17^ and 3.4%.^3^ Many prefer pills on injectables,^5^ 82%,^17^ 49%(36) 16.8%^16^ and 13.7%^19^ used OCs; 3.4% utilized injectables.^3^ Being safe and efficient, IUCDs is another common mode,^3^,^21^ used by 15.5%^21^ and 13% of women worldwide.^15^ Unmarried and nullipara women avoid this method,^7^ while multipara with more live issues prefer it.^21^

In this study, there was no report of male-ligation/vasectomy. Female sterilization is now more commonly used in the world,^20^ including Pakistan^6^ and India,^22^ yet seldom used by unmarried ladies. In India, it was commoner among ladies who got married at early age, are poor or had borne more than desired children.^22^

Religious, social, cultural and socio-economic factors; all affect type and frequency of use of contraceptive methods in the world; especially in Asia^1^ including Pakistan^4^ and India.^22^ This study validated significant relationship between type of contraception used and client’s age, education, number of children, religion, socioeconomic class, religious beliefs about use of contraception and husband’s education, but there was no considerable pressure on the respondents from husband’s side. Researchers documented that contraception was affected by age at first sex, sexual partners, marital status, education of woman and her partner, parity, live issues and desire for pregnancy^16^ Although females illustrated more knowledge and inclination, nevertheless, partners played a pivotal role in decision making regarding contraception in the world including Pakistan^4^,^23^ & Sri-Lanka.^24^ In this study, 35.2% declared contraception to be irreligious; especially Muslims. In Pakistan 70% believed Islam did not oppose contraception.^6^ Illiterate, poorer, rural and younger, poorer and less literate women depicted more acceptability towards contraception, although they may adapt the traditional and economical methods like condoms^25^ especially in Pakistan,^4^,^23^ while in Sri-Lanka, richer, more literate, Christian/Buddhist were more compliant towards family planning than poorer, less educated and Muslim/Hindu ladies.^24^

This study has highlighted the prevalence of use of various contraceptive techniques in respondents from a major city of Pakistan. The impact of customs/traditions on family planning; along with various demographic features could not be ruled out. This research is unique and stands at its own being first of its type, as no similar study has yet been carried out in this region on the subject matter as per the skimpy awareness of the authors.

### Limitations of the study

Although the sample size was statistically enough to represent population of the concerned city, nevertheless, a bigger cohort from more versatile areas of the city could represent similar population more accurately. Respondents included limiters reporting to hospital for advice/prescription, who could be more health conscious, thus, selection bias was inevitable. Ladies residing in streets and slums of the city could definitely alter the findings. Demographic forms and questions were self-designed, so incomplete data collection and interviewer bias could not be ruled out.

### Directions for future research

Awareness, education and availability of contraceptive devices must attenuate under-utilization of contraception.

## CONCLUSION

Trends of use of contraceptive methods are substantially affected by multiple demographic factors, and religious/social beliefs and myths. Adequate family planning can only be enhanced by adapting multidisciplinary approach, engaging ladies as well as their spouses, addressing all misconceptions and misperceptions. The under-privileged populations must be approached to be equally benefitted from the technology.
